# Social factors and chronic pain: the modifying effect of sex in the Stockholm Public Health Cohort Study

**DOI:** 10.1093/rheumatology/keab528

**Published:** 2021-07-08

**Authors:** Jesús Prego-Domínguez, Eva Skillgate, Nicola Orsini, Bahi Takkouche

**Affiliations:** 1 Department of Preventive Medicine, University of Santiago de Compostela, Santiago de Compostela, Spain; 2 Musculoskeletal & Sports Injury Epidemiology Center, Institute of Environmental Medicine, Karolinska Institutet; 3 Naprapathögskolan-Scandinavian College of Naprapathic Manual Medicine; 4 Department of Health Promotion Science, Sophiahemmet University; 5 Department of Public Health Sciences, Karolinska Institute, Stockholm, Sweden; 6 Centro de Investigación Biomédica en Red de Epidemiología y Salud Pública (CIBER-ESP), Madrid, Spain

**Keywords:** socio-economic status, sociological factors, occupational stress, job strain, chronic pain

## Abstract

**Objectives:**

To assess the relationship between social factors (socio-economic status, household load and job strain) and chronic pain occurrence, and the role of gender in this relationship.

**Methods:**

We used data corresponding to 8 years of follow-up of the Stockholm Public Health Cohort Study (2006–2014) to compute Adjusted Incidence Rate Ratios (IRRs) and additive interaction measures of chronic pain episodes, social factors, and sex in 16 687 subjects.

**Results:**

For men, increased rates of chronic pain occurrence were observed for skilled workers (IRR = 1.27, 95% CI: 0.99, 1.61) and lower non-manual employees (IRR = 1.37, 95% CI: 1.05, 1.78), compared with unskilled workers; subjects with high household load (IRR = 1.39; 95% CI: 1.03, 1.88), compared with those with a null score; and subjects with active jobs (IRR = 1.27, 95% CI: 1.06, 1.51), compared with those with low-strain jobs. For women, we observed decreased rates of chronic pain occurrence in lower (IRR = 0.82, 95% CI: 0.68, 0.99), intermediate (IRR = 0.74, 95% CI: 0.63, 0.88) and higher non-manual employees (IRR = 0.65, 95% CI: 0.54, 0.79), compared with unskilled workers. Compared with subjects with a null score, women with low household load showed a lower rate of chronic pain occurrence (IRR = 0.85; 95% CI: 0.72, 1.00). Compared with subjects with low-strain jobs, those with passive jobs (IRR = 1.21; 95% CI: 1.02, 1.44) and high-strain jobs (IRR = 1.46; 95% CI: 1.02, 2.09) showed higher rates of chronic pain occurrence.

**Conclusion:**

In general, our analysis yielded different, if not opposite, results when data were stratified by sex. Sex may then represent an effect modifier of the relationship between social factors and chronic pain.

Rheumatology key messages

- Low socio-economic status and a high-job-strain household load are related to chronic pain occurrence.
- Sex is an effect modifier of the relationship between socio-economic status and chronic pain; that is, this relationship is different between men and women.

## Introduction

Chronic pain (CP) represents an important public health issue worldwide. The prevalence of moderate to severe non-cancer CP is 19% in Europe [1], while 1 out of 10 people in the world is newly diagnosed every year with this syndrome [2]. CP has a large impact on daily life: low-back and neck pain are among the five leading causes of disability-adjusted life years (DALYs), and the remaining causes, such as major depression and cardiovascular disease, are all associated with CP [3]. Furthermore, the costs of CP are estimated at $635 billion per year in the USA and €200 billion per year in Europe, more than those of cancer and cardiovascular diseases together [4, 5]. Although its distribution presents some variations across the globe, CP strongly affects all populations, regardless of age, race, sex, income or country, which has led some authors and the International Association for the Study of Pain (IASP) to describe it as a ‘global crisis’ [2].

In Sweden, the prevalence of CP is similar to that in other regions of Europe, with an overall 18% prevalence of non-cancer moderate-to-severe CP, though reaching 54.7% for general CP in some specific areas [6]. The cost of the syndrome has been estimated for the whole country at €32 billion per year, that is, one 10th of the Swedish Gross Domestic Product [7].

Despite its relevance as a public health issue, epidemiologic research on CP as a condition on its own is scarce, as it only began to be addressed from this perspective within the last decade . Until recently, only specific anatomic location pain (such as low-back pain) or specific pain syndromes (such as chronic widespread pain) were investigated. However, female sex, social factors and job environment have been consistently associated with the onset and severity of both CP and CP-related syndromes in epidemiologic studies [5].

Sex is one of the variables most strongly associated with CP [8]. Women present a higher prevalence of CP syndromes, such as FM, migraine or temporomandibular disorders, as well as higher pain intensity [9].

Furthermore, socio-economic status (SES) is shown to be inversely related to CP [10], and economic disadvantage was linked to increased CP-related disability [11]. However, most of the evidence is based on research with cross-sectional designs of questionable value for causal inference purposes and on specific pain conditions only.

In a similar fashion, occupational psychosocial factors were related to the occurrence of new episodes of CP, as well as to their persistence over time, albeit mainly in the context of musculoskeletal disorders [12, 13]. Less is known about the role of household social factors, such as housework load, social support, or care of dependent relatives, although some studies have suggested a relationship between these factors and higher prevalence and severity of pain [14].

In this study, we aimed at elucidating the role of SES, and household and job-related factors in the occurrence of CP, using data from the Stockholm Public Health Cohort (SPHC), an 8-year follow-up study covering a large population from the Stockholm Council area. We also aimed at assessing whether the effect of those social factors on CP was modified by sex, i.e. whether the effect is similar among men and among women.

## Methods

### Study population and questionnaire

This study is based on the 2006 wave of the SPHC, a cohort study reporting data from a random sample of the Stockholm City County population, an urban region with ∼1.4 million inhabitants. The Stockholm Public Health Cohort (SPHC) is a population-based cohort study that has been established within the framework of public health surveys that took place in 2002, 2006, 2010 and 2014. It is based on a mix of questionnaire and register data. Data from the four surveys have been pooled, and participants (*n* = 115 000, aged ≥18 years)were followed up longitudinally for health, lifestyle and social outcomes. Self-reported data were supplemented with information from the Swedish health system and administrative registers, which contained data on study participants as well as on their first-degree relatives. The SPHC assesses a wide range of exposure and health outcomes. The overall response rate of each subcohort was at least >70% [15]. The constitution of the cohort was approved by the Stockholm Regional Ethical Review Board (Dnr 2010/1879–31/5; Dnr 2007/545–31).

In our study, postal and web-based questionnaires were sent in 2006 to 56 634 randomly selected participants aged 18–84 years, after stratification by sex and residential area. At baseline, 34 707 subjects (61%) answered the questionnaire, 40 of which were excluded due to the lack of identification number. Subjects reporting CP at baseline (*n* = 11 586) were excluded from the follow-up.

CP status was assessed at baseline using three questions from the 2006 questionnaire, which determined whether, in the last 6 months, subjects had pain in their neck, shoulders or arms, low back pain, and/or pain in their hips, thighs or knees. The five possible answers were: ‘No’, ‘Yes, a few days in the past 6 months’, ‘Yes, a few days per month’, ‘Yes, a few days per week’ and ‘Yes, every day’. Subjects without regular pain in the previous 6 months were included in the follow-up and formed the baseline population, which was followed for 8 years.

### Outcome definition

The outcome was defined as CP at any location, measured by the questions ‘In the past 6 months did you have pain in the upper back or neck/low back/shoulder or arms?’ CP status was defined as having regular pain in the past 6 months. The case definition was established according to the IASP definition [16].

### Exposure definition

#### Socio-economic status

We used the *Swedish Socioeconomic Classification,* developed by Statistics Sweden, which classifies individuals into six socio-economic categories, according to their current (or previous for non-working people) occupational status: unskilled workers, skilled workers, lower non-manual employees, intermediate non-manual employees, higher non-manual employees and self-employed [17]. Unemployed subjects with no previous work experience were considered as having missing values in this variable. They represented 221 persons out of a study population of 23 081 (i.e. <1%).

#### Household load

We assessed the total household social load using the following elements: shared household with children aged <12 years, hours per week of domestic work (excluding occupational work), hours per week dedicated to the care of elderly relatives, and social support.

To assess child care, we used the question ‘If you live together with children, what is their age?’ The possible answers were ‘0–5’ and ‘6–12’. The score was ‘1’ if one of them was answered, ‘2’ if both, and ‘0’ if none of them was selected.

The time spent working at home was defined by the question ‘How many hours per week do you spend working at home?’, scoring ‘1’ if it was ≤10 h, ‘2’ if it was 11–20 h and ‘3’ if it was ≥21 h.

To assess whether a subject had relatives to care for, we used answers to two questions ‘Do you have an ill or elderly relative whom you help with everyday chores, watch or care for?’ (No/Yes), and ‘If yes, on average how many hours of work does this mean for you per week?’ We scored as ‘0’ those who responded ‘No’ to the first question. For those who answered ‘Yes’, we scored ‘1’ those who answered <6 h per week and ‘3’ those who answered 6 or more hours per week.

Lack of social support was assessed using the questions ‘Do you know any people who can provide you with personal support for personal problems or crises in your life?’ and ‘Can you obtain help from anyone in the event of illness or practical problems?’, scoring each as ‘1’ if the answer was ‘No, never’ or ‘No, usually not’ and ‘0’ if the answer was ‘Yes, always’ or ‘Yes, for the most part.’

#### Job strain

We assessed psychosocial exertion at work using four questions included in the baseline questionnaire, as previously reported [12]. Briefly, we dichotomized job demands and job control into high and low, to obtain four categories: low strain (low job demands and high job control), active job (high job demands and high job control), passive job (low job demands and low job control) and high job strain (high job demands and low job control). This model has been shown to have high internal consistency and high reliability [18].

### Confounding assessment

The following covariates were assessed as potential confounders: sex, age, BMI, long-term illness, trouble sleeping, comorbidity (diabetes, asthma, lung disease, RA, depression, chronic fatigue/burnout), physical activity, smoking status, alcohol consumption, perceived stress level, physical exertion at work in the past 12 months, whether or not family life was negatively affected by job demands, psychological distress (as per General Health Questionnaire GHQ-12) [19] and economic distress. Those covariates that changed Adjusted Incidence Rate Ratio (IRR) estimates of SES by >10% were introduced in the final model [20].

### Statistical analysis

Person-time was calculated from the date of administration of the baseline questionnaire until the onset of CP, loss to follow-up or end of the study, whichever came first. Assuming constant incidence during that period of time, cases of CP were assigned half of the period between the last follow-up and the onset of the disease.

Additive interaction analyses between sex and each exposure factor were performed [21]. Variables were considered binary, with the level with the lowest risk of CP as a reference category [22]. For each sex-exposure interaction, we computed the adjusted Relative Excess Risk due to Interaction (RERI), also named Interaction Contrast Ratio (ICR), the Attributable Proportion (AP), and the Synergy index (S) along with their 95% CIs for each interaction [21, 22].

We used Poisson regression to estimate IRRs of CP and their 95% CIs for social factors. The analysis of SES was finally adjusted for age and home/family life negative affect by job demands. The analyses of household load and job strain were adjusted for age and perceived stress.

All analyses were performed with STATA/MP software version 15.1 (Stata Corp LLC, Tx, USA).

### Robustness analyses

To assess the effect of attrition in our study, we carried out the following three analyses. In the first analysis, we used multiple imputations by chained equations with 20 imputed datasets, and then repeated each of the Poisson regression analyses carried out previously [23].

Baseline socio-economic data were marginally associated with attrition. We, therefore, corrected for differential attrition in a second analysis in which we used Inverse Probability Weighting to recalculate our estimates. We used logistic regression models with baseline variables to calculate the predicted probability of follow-up completeness. The inverse of this probability was used as a weight in subsequent Poisson regression models [24].

In the third analysis, we recalculated the observed IRRs in two extreme scenarios. We first assumed that all participants lost to follow-up developed CP, and subsequently we assumed that none of those participants developed the disease.

## Results

Baseline characteristics of the study population are presented in Table 1. A total of 23 081 individuals (11 311 men and 11 770 women) were free of CP at the beginning of the study and were then included in the follow-up. According to the stratification of the sample, the population was evenly distributed across sex and age groups, with a mean age of 46.83 and 45.28 years for men and women, respectively. Non-manual employees’ categories accounted for nearly two-thirds of the sample, compared with the unskilled and skilled workers categories, which, per the Swedish Socioeconomic Classification, include manual employees and self-employed workers. The majority of the population had low household load scores and low job strain. The distribution of these factors was similar for men and women.

**Table 1:** Characteristics of the study population (*n* = 16 687) of the Stockholm Public Health Cohort

Variable	Total population		Men		Women	
	*n*	%	*n*	%	*n*	%
Gender						
Men	7892	47.3	–	–	–	–
Women	8795	52.7	–	–	–	–
Age						
18–35	4386	26.3	1811	22.9	2575	29.3
36–47	4078	24.4	1901	24.1	2177	24.7
48–61	4356	26.1	2168	27.5	2188	24.9
62–84	3867	23.2	2012	25.5	1855	21.1
Socio-economic status						
Unskilled workers	1981	13.0	930	12.8	1051	13.2
Skilled workers	1603	10.5	882	12.2	721	9.0
Lower non-manual employees	2226	14.6	626	8.6	1600	20.1
Intermediate non-manual employees	4137	27.2	1808	24.9	2329	29.2
Higher non-manual employees	3855	25.3	2098	28.9	1757	22.0
Self-employed	1416	9.3	904	12.5	512	6.4
Household load score^a^						
0	2225	13.3	1127	14.3	1098	12.5
Low	9896	59.4	4848	61.6	5048	57.4
Medium	3820	22.9	1654	21.0	2166	24.6
High	721	4.3	245	3.1	476	5.4
Job strain type						
Low job strain	9668	80.4	4596	82.1	5072	78.9
Active job	1429	11.9	634	11.3	795	12.4
Pasive job	797	6.6	315	5.6	482	7.5
High job strain	130	1.1	50	0.9	80	1.2

a Household load scoring system: shared household with children aged <12 years (0–2 points), h per week of domestic work, excluding occupational work (1–3 points), h per week dedicated to the care of elderly relatives (0–3 points), social support (1 point). Total scoring: low = 1–2, medium = 3–4, high = ≥5.

A total of 6394 (27.7%) individuals from the baseline population did not report any information on pain status during the follow-up. The final population included 16 687 subjects.

We observed 4107 new CP cases during the follow-up, representing 98 122 person-years, which yielded an overall incidence rate of 0.041 year^−1^ (0.035 year^−1^ for men and 0.047 year^−1^ for women).

Interaction analyses (Table 2) yielded moderate positive additive interaction between female sex and SES (RERI = 0.36, 95% CI: 0.13, 0.59) and a tendency to positive interaction between female sex and job strain (RERI = 0.17, 95% CI: −0.84, 1.19). No interaction was found between sex and household load (RERI = −0.11, 95% CI: −0.52, 0.29). The results of the AP and S statistics confirmed these findings.

**Table 2:** Measures of additive interaction between gender and social factors in the Stockholm Public Health Cohort

Interaction	Adjusted chronic pain IRR (95% CI)	RERI (95% CI)	AP (95% CI)	S (95% CI)
Socio-economic status^a^/gender		0.35 (0.12, 0.58)	0.21 (0.08, 0.33)	1.98 (0.89, 3.06)
Men with high socio-economic status	1 (ref)			
Women with high socio-economic status	1.26 (1.16, 1.38)			
Men with low socio-economic status	1.09 (0.96, 1.26)			
Women with low socio-economic status	1.72 (1.51, 1.92)			
Household load score^b^/gender		−0.12 (−0.53, 0.28)	−0.08 (−0.36, 0.19)	0.80 (0.24, 1.36)
Men with high score	1.33 (1.05, 1.70)			
Women with high score	1.50 (1.25, 1.76)			
Men with low score	1 (ref)			
Women with low score	1.29 (1.21, 1.38)			
Job strain level^b^/gender		0.18 (−0.84, 1.20)	−0.09 (−0.41, 0.59)	1.23 (−0.25, 2.71)
Men with high job strain	1.49 (0.89, 2.48)			
Women with high job strain	1.96 (1.27, 2.65)			
Men with low job strain	1 (ref)			
Women with low job strain	1.29 (1.20, 1.39)			

a Adjusted for age and home/family affect by job demands; ^b^adjusted for age and perceived stress. Scoring system: Socio-economic status: low = unskilled/skilled manual workers, high = non-manual employees/self-employed; Household load: low = low-strain/active job/passive job, high = high job strain. IRR: Incidence Rate Ratio; RERI: Relative Excess Risk due to Interaction; AP: Attributable Proportion; S: Synergy Index; ref.: reference.

In general, our analysis yielded different results, if not opposite, when data were stratified by sex (Table 3). For SES, we observed that male skilled workers (IRR = 1.30, 95% CI: 1.03, 1.66) and lower non-manual male employees (IRR = 1.29, 95% CI: 1.00, 1.66) have higher rates than male unskilled workers, while the results of the rest of categories were compatible with no increase in the CP rate. On the contrary, among women, skilled workers (IRR = 0.85, 95% CI: 0.69, 1.03) and lower (IRR = 0.76, 95% CI: 0.64, 0.90), intermediate (IRR = 0.70, 95% CI: 0.60, 0.82) and higher non-manual employees (IRR = 0.60, 95% CI: 0.50, 0.71) showed a decrease in the rates when compared with unskilled female workers.

**Table 3:** IRRs and 95% CIs of chronic pain in relation to social factors in the Stockholm Public Health Cohort

	Men			Women		
	No. of cases (*N*)/years at risk	Crude IRR (95% CI)	Adjusted IRR^a^ (95% CI)	No. of cases (*N*)/years at risk	Crude IRR (95% CI)	Adjusted IRR^a^ (95% CI)
Socio-economic status						
Unskilled workers	165/506	1	1	333/1058	1	1
Skilled workers	208/720	1.36 (1.11, 1.67)	1.30 (1.02, 1.66)	208/648	0.89 (0.75, 1.06)	0.85 (0.69, 1.03)
Lower non-manual employees	151/534	1.35 (1.08, 1.68)	1.29 (1.00, 1.66)	431/1442	0.79 (0.68, 0.91)	0.76 (0.64, 0.90)
Intermediate non-manual employees	382/1312	1.12 (0.93, 1.34)	0.97 (0.78, 1.19)	620/2124	0.76 (0.67, 0.87)	0.70 (0.60, 0.82)
Higher non-manual employees	449/1518	1.15 (0.96, 1.37)	0.97 (0.79, 1.20)	442/1444	0.71 (0.61, 0.82)	0.60 (0.51, 0.71)
Self employed	206/656	1.24 (1.01, 1.53)	1.06 (0.84, 1.35)	156/472	0.92 (0.76, 1.11)	0.86 (0.69, 1.07)
Household load score^b^						
0	265/890	1	1	303/958	1	1
Low	1010/3368	0.87 (0.76, 0.99)	1.04 (0.89, 1.23)	1348/4480	0.92 (0.82, 1.05)	0.81 (0.70, 0.94)
Medium	337/1154	0.83 (0.71, 0.98)	0.98 (0.81, 1.19)	616/1964	1.01 (0.88, 1.15)	0.84 (0.71, 0.99)
High	71/230	1.29 (1.00, 1.69)	1.42 (1.07, 1.90)	153/478	1.19 (0.98, 1.45)	0.95 (0.76, 1.18)
Job strain level						
Low strain	917/3134	1	1	1312/4364	1	1
Active job	174/544	1.43 (1.22, 1.69)	1.23 (1.04, 1.46)	247/806	1.25 (1.09, 1.43)	1.06 (0.92, 1.22)
Passive job	59/194	0.98 (0.75, 1.27)	0.98 (0.75, 1.28)	151/494	1.29 (1.09, 1.52)	1.21 (1.02, 1.43)
High strain	15/58	1.65 (0.99, 2.76)	1.62 (0.97, 2.70)	32/84	1.79 (1.26, 2.54)	1.55 (1.09, 2.20)

a Socio-economic status adjusted for age and home/family affect by job demands; household load and job strain adjusted for age and perceived stress. ^b^Household load scoring system: shared household with children aged <12 years (0–2 points), h per week of domestic work, excluding occupational work (1–3 points), h per week dedicated to the care of elderly relatives (0–3 points), social support (1 point). Total scoring: low = 1–2, medium = 3–4, high = ≥5. IRR: Incidence Rate Ratio.

A similar difference in the effect between men and women was observed for household load score. Among men, subjects with a high household score showed a higher rate than subjects with a score of 0 (IRR = 1.42, 95% CI: 1.07, 1.90). Among women, when compared with subjects with a null score, people with a low (IRR = 0.81, 95% CI: 0.70, 0.94) and intermediate (IRR = 0.84, 95% CI: 0.71, 1.00) household score showed a decrease in the rate, while the results for the high score category were compatible with no change in the rates of CP.

As for job strain level, male subjects with active jobs showed a higher rate than subjects with low-strain jobs (IRR = 1.23, 95% CI: 1.04, 1.46). The rest of categories seemed to be unrelated to the occurrence of CP among men. Among women, we observed that subjects with passive jobs (IRR = 1.21, 95% CI: 1.02, 1.44) and high-strain jobs presented higher rates than subject with low-strain jobs (IRR = 1.54, 95% CI: 1.08, 2.19), while the results of the active job category did not support any increase in the rates.

The results from the robustness analyses, in which the effect of attrition was assessed, did not differ substantially from those obtained in the original analyses (Supplementary Table S1, available from *Rheumatology* online). In particular, no point estimate in the Inverse Probability Weighting and in the Multiple Imputation procedures showed a deviation of ≥20% from the uncorrected point estimates. The results of the analysis of extreme scenarios showed very moderate changes. The most noticeable change was found in the additive interaction measure of job strain level, in which RERI changed from a slight positive interaction (0.18) to no interaction (−0.04) when we assumed that none of the participants lost to follow-up developed CP, and in the point estimates corresponding to the highest level of household load (from 1.43 to –0.94) and job strain (from 1.62 to –1.12) in men, when we assumed that all subjects lost to follow-up presented the outcome.

## Discussion

The aim of this study was to elucidate the relationship of CP to the main social factors, including socio-economic, household and job status, and to assess whether this relationship was different between men and women. We considered CP as a homogeneous condition, and we used prospective data from a large cohort representative of the population of the Stockholm Council area.

Our results suggest that sex is a moderate effect modifier of the relationship between social factors and CP. We found evidence for a departure from additivity of the relationship between sex and SES, and sex and job strain, supporting a moderate increase in CP incidence rate in women exposed to high levels of these social factors. Intriguingly, men with low-to-intermediate levels of SES reported higher incidence of CP. The differences in frequency and severity of CP between men and women have been mentioned in previous research. Women tend to report more severe bouts of CP and present a higher prevalence [11]. This feature remains constant across different SES levels, ages and countries, including Sweden [25].

Furthermore, in previous research, it has been shown that social factors and both CP onset and CP disability are related [26]. An inverse relationship between SES and CP, such as the one observed in our study, has already been reported in longitudinal and cross-sectional studies. In a recent global population survey, sex, low education and low wealth were found to be strongly associated with higher back-pain prevalence and related disability [27], while a recent British birth cohort study showed that SES was associated with chronic widespread pain at the age of 45 [28]. These studies did not report specific results by sex.

Both animal and human research have highlighted differences between males and females regarding nociception, pain threshold and induced analgesia, probably due to differences in hormonal and opioid receptors [29]. Several mechanisms can explain these differences: first, oestrogen and progesterone are known to interact with glial cells, and, second, differences between men and women have been found in the serotonin/dopamine system, with a prominent serotonic state in males [30]. Furthermore, studies carried out on transsexuals who received cross-sex hormones have shown that intake of female gonadal hormones increases pain sensitivity and the probability of occurrence of CP syndromes, while androgens and testosterone exert a protective effect against pain [31]. Genetic mechanisms were also invoked. Indeed, the genes that encode Catechol-O-methyltransferase (*COMT*), GTP cyclohydrolase and Mu-opioid receptor, an important opioid binding site, were found to have different effects depending on gender [30].

In addition to hormonal and genetic mechanisms, psychosocial factors (including pain coping, exposure to stress and gender roles) may also explain sex differences in pain occurrence.

The relationship between household workload and CP was infrequently described in the literature. A modest increase in the prevalence of back and neck pain was found in subjects with heavy load in a European cross-sectional study [32], while another Japanese study of similar design reported a higher frequency of pain among caregivers of persons with dementia [14]. Furthermore, a meta-analysis of 22 studies reported a higher risk of musculoskeletal disorders among subjects with high job strain [33], while the risk of other specific pain conditions was also found to be increased in other studies [12, 18].

The causal mechanisms that connect job strain, and presumably high household workload, to CP onset seem to be related to the interaction between physical load and stress caused by psychosocial work factors such as low job control or high job demands [33]. However, it is remarkable that, in our study, adjusting for stress did not modify the relationship of CP to job strain or household workload. Animal and human neuroimaging studies have suggested that severe or moderate chronic stress could lead to dysregulation in the hypothalamic–pituitary–adrenal (HPA) axis that would cause a neuroinflammatory state that has been linked to several CP syndromes (such as neuropathic pain, FM and chronic back pain) [34, 35].

In our study, we cannot exclude some potential for selection bias due to attrition, despite the results of our extensive robustness assessment. Indeed, subjects who failed to complete their follow-up were slightly younger, included a larger proportion of men and of individuals with no household load, and belonged more frequently to the unskilled workers’ category than full respondents. Low SES is consistently related to a higher risk of CP in previous studies, while incidence of CP remains constant through age [5]. Thus, if bias exists due to non-response in these groups, it would distort our results towards the null value. The true effect is then probably stronger than the effect observed in our study. Furthermore, male sex has been related to a lower risk of CP in former studies [29], while scarce evidence is available on the effect of household load on CP. Therefore, a certain degree of risk overestimation cannot be ruled out.

Finally, we found a low proportion of subjects with high household load and high job strain in our study population. However, this low proportion did not affect the precision of our estimates as, except for men with high job strain, the estimates for these categories were significant.

Studies that determine CP incidence are scarce due to the difficulties in carrying out longitudinal studies [36]. While the CP incidence found in our study (0.041 year^−1^) was similar to that of another recent Swedish study (0.054 year^−1^) [37], it was only half of that found in a British study (0.083 year^−1^) [38]. This relatively low incidence could be explained by the fact that our baseline questionnaire assessed pain located in the upper back or neck, low back, and shoulder or arms only. This case definition excluded CP syndromes, such as neuropathic pain or migraines that affect other body locations, and this represents a limitation of our study.

Theoretically, exposure variables may have varied through time, but we believe that a certain lag time should elapse between exposure to a given social factor and onset of acute pain, first, then onset of CP. The effect of this exposure on the outcome is not immediate. Also, SES and the other social factors of our study are not transient exposures, but rather factors that are stable over time. Measurement of the exposure at baseline, instead of measurement during follow-up, is then the most germane assessment.

As a conclusion, to our knowledge, ours is the first study based on longitudinal data from a large cohort that assesses the relationship of social factors to the risk of CP. Our results support evidence for a relationship of common modifiable social factors (such as SES, household work and job strain) to CP that cannot easily be explained by confounding by other factors. The fact that sex is an important modifier of this relationship is provocative and strongly warrants attention in future research.

##  

*Funding:* The Regional Ministry of Education, Universities and Vocational Training, Santiago de Compostela, Spain, contributed to the funding for this study (ED431C 2018/20).

*Disclosure statement*: The authors have declared no conflicts of interest.

## Data availability statement

The datasets generated during and/or analysed during the current study are available from the corresponding author on reasonable request or may be uploaded to a repository.

## Supplementary data

Supplementary data are available at *Rheumatology* online.

## Supplementary Material

keab528_Supplementary_Data
