# Supplementary material for: Social factors and chronic pain: the modifying effect of sex in the Stockholm Public Health Cohort Study
Source: Rheumatology (Oxford). 2021 Jul 8;61(5):1802–9. doi: 10.1093/rheumatology/keab528 (PMC9071550; doi:10.1093/rheumatology/keab528)
Supplement: keab528_Supplementary_Data [file keab528_supplementary_data.docx]

| **Supplementary Table S1**: Incidence Rate Ratios (IRR) and 95% Confidence Intervals (CI) of the Robustness Analyses of Chronic Pain in Relation With Social Factors in the Stockholm Public Health Cohort. | | | | | | | | | | | | | |  |
| --- | --- | --- | --- | --- | --- | --- | --- | --- | --- | --- | --- | --- | --- | --- |
|  | | | Men | | | | | |  | Women | | | | |
|  | | | Observed | | Multiple imputation | Inverse Probability Weighting | Lost subjects considered cases | Lost subjects considered non cases |  | Observed | Multiple imputation | Inverse Probability Weighting | Lost subjects considered cases | Lost subjects considered non cases |
| **Socioeconomic status^a^** | | |  | |  |  |  |  |  |  |  |  |  |  |
| Unskilled workers | | | 1 | | 1 | 1 | 1 | 1 |  | 1 | 1 | 1 | 1 | 1 |
| Skilled workers | | | 1.30 (1.02, 1.66) | | 1.17 (0.94, 1.45) | 1.28 (0.99, 1.65) | 1.05 (0.93, 1.18) | 1.39 (1.09, 1.76) |  | 0.85 (0.69, 1.03) | 0.91 (0.75, 1.10) | 0.83 (0.67, 1.03) | 0.91 (0.80, 1.03) | 0.91 (0.75, 1.11) |
| Lower non-manual | | | 1.29 (1.00, 1.66) | | 1.17 (0.93, 1.47) | 1.31 (1.00, 1.73) | 0.98 (0.85, 1.12) | 1.49 (1.15, 1.92) |  | 0.76 (0.64, 0.90) | 0.85 (0.72, 1.01) | 0.70 (0.59, 0.85) | 0.80 (0.72, 0.90) | 0.90 (0.76, 1.07) |
| Intermediate non-manual | | | 0.97 (0.78, 1.19) | | 0.96 (0.80, 1.16) | 0.99 (0.79, 1.25) | 0.81 (0.73, 0.91) | 1.19 (0.97, 1.48) |  | 0.70 (0.60, 0.82) | 0.81 (0.69, 0.94) | 0.70 (0.59, 0.82) | 0.77 (0.69, 0.85) | 0.87 (0.75, 1.02) |
| Higher non-manual | | | 0.97 (0.79, 1.20) | | 0.98 (0.83, 1.17) | 0.99 (0.79, 1.24) | 0.82 (0.73, 0.91) | 1.20 (0.98, 1.48) |  | 0.60 (0.51, 0.71) | 0.72 (0.62, 0.85) | 0.62 (0.52, 0.74) | 0.67 (0.60, 0.75) | 0.79 (0.67, 0.93) |
| Self employed | | | 1.06 (0.84, 1.35) | | 1.03 (0.84, 1.27) | 1.06 (0.82, 1.37) | 0.97 (0.85, 1.10) | 1.14 (0.90, 1.44) |  | 0.86 (0.69, 1.07) | 0.95 (0.78, 1.16) | 0.87 (0.69, 1.10) | 0.89 (0.77, 1.04) | 1.00 (0.80, 1.24) |
|  | | |  | |  |  |  |  |  |  |  |  |  |  |
| **Household load score^b^** | | |  | |  |  |  |  |  |  |  |  |  |  |
| 0 | | | 1 | | 1 | 1 | 1 | 1 |  | 1 | 1 | 1 | 1 | 1 |
| Low | | | 1.04 (0.89, 1.23) | | 1.04 (0.89, 1.21) | 1.02 (0.86, 1.21) | 0.82 (0.74, 0.92) | 1.31 (1.11, 1.54) |  | 0.81 (0.70, 0.94) | 0.92 (0.79, 1.07) | 0.80 (0.69, 0.94) | 0.74 (0.67, 0.83) | 1.05 (0.91, 1.23) |
| Medium | | | 0.98 (0.81, 1.19) | | 0.98 (0.82, 1.17) | 0.96 (0.79, 1.17) | 0.81 (0.72, 0.91) | 1.24 (1.03, 1.50) |  | 0.84 (0.71, 0.99) | 0.95 (0.81, 1.12) | 0.84 (0.71, 1.00) | 0.77 (0.68, 0.86) | 1.10 (0.93, 1.31) |
| High | | | 1.43 (1.07, 1.90) | | 1.24 (0.94, 1.64) | 1.39 (1.03, 1.89) | 0.94 (0.79, 1.12) | 1.73 (1.30, 2.31) |  | 0.95 (0.76, 1.18) | 1.03 (0.83, 1.26) | 0.92 (0.73, 1.15) | 0.84 (0.72, 0.97) | 1.18 (0.95, 1.46) |
|  | | |  | |  |  |  |  |  |  |  |  |  |  |
| **Job strain level^b^** | | |  | |  |  |  |  |  |  |  |  |  |  |
| Low strain | | | 1 | | 1 | 1 | 1 | 1 |  | 1 | 1 | 1 | 1 | 1 |
| Active job | | | 1.23 (1.04, 1.46) | | 1.16 (0.99, 1.36) | 1.24 (1.03, 1.49) | 1.06 (0.95, 1.18) | 1.30 (1.09, 1.54) |  | 1.06 (0.92, 1.22) | 1.06 (0.92, 1.22) | 1.10 (0.95, 1.28) | 1.02 (0.92, 1.13) | 1.09 (0.94, 1.25) |
| Passive job | | | 0.98 (0.75, 1.28) | | 0.99 (0.79, 1.24) | 0.96 (0.72, 1.28) | 1.05 (0.92, 1.19) | 0.89 (0.68, 1.16) |  | 1.21 (1.02, 1.43) | 1.15 (0.97, 1.35) | 1.16 (0.96, 1.40) | 1.11 (0.99, 1.24) | 1.16 (0.98, 1.38) |
| High strain | | | 1.62 (0.97, 2.70) | | 1.39 (0.86, 2.23) | 1.34 (0.77, 2.34) | 1.12 (0.80, 1.57) | 1.61 (0.96, 2.69) |  | 1.55 (1.09, 2.20) | 1.31 (0.93, 1.84) | 1.57 (1.07, 2.32) | 1.35 (1.06, 1.72) | 1.34 (0.95, 1.91) |
|  |  |  | |  | | | | | | | | | |  |
| ^a^ adjusted for age and home/family affect by job demands; **^b^** adjusted for age and perceived stress. | | | | | | | | | | | | | |  |
| Household load scoring system: shared household with children aged <12 years (0-2 points), hours per week of domestic work, excluding occupational work (1-3 points), hours per week dedicated to the care of elderly relatives (0-3 points), social support (1 point). Total scoring: low = 1-2, medium = 3-4, high = ≥ 5. | | | | | | | | | | | | | |  |
